# Effectiveness of Periarticular Local Infiltration Analgesia in Primary Total Knee Arthroplasty: A Retrospective Case Series

**DOI:** 10.7759/cureus.107786

**Published:** 2026-04-27

**Authors:** Mehdi Nabil, K Said, Youssef Qamouss, Belkacem Chagar

**Affiliations:** 1 Anesthesiology and Reanimation, Military Hospital of Avicenne, Marrakech, MAR; 2 Anesthesia and Critical Care, Military Hospital of Avicenne, Marrakech, MAR; 3 Orthopaedics and Traumatology, Military Hospital of Avicenne, Marrakech, MAR

**Keywords:** enhanced recovery, local infiltration analgesia, periarticular infiltration, postoperative pain, total knee arthroplasty

## Abstract

Background

Periarticular local infiltration analgesia (LIA) is widely used within multimodal and enhanced recovery after surgery (ERAS)-based pathways for total knee arthroplasty (TKA). However, real-world data remain heterogeneous, particularly regarding simplified injection regimens without adjuvants. This study aimed to describe early postoperative outcomes following a standardized periarticular LIA protocol in primary TKA.

Methods

This retrospective single-center case series included 30 consecutive patients undergoing primary TKA for knee osteoarthritis between January and June 2025. All patients received spinal anesthesia and a standardized three-stage periarticular infiltration protocol using diluted bupivacaine and lidocaine without adjuvants. Outcomes were analyzed descriptively and included visual analog scale (VAS) pain scores, documented opioid administration, time to mobilization, length of stay, complications, and patient satisfaction.

Results

The mean age was 72.2±7.3 years, and 73.3% of patients were women. Postoperative pain, assessed using the visual analog scale (VAS, 0-10), demonstrated a progressive decrease over the first 48 hours following surgery. At six hours postoperatively, the mean VAS score was 2±1 for all patients (n=30). At 12 hours, the mean VAS increased to 4±1, followed by a reduction to 3±1 at 24 hours, and further decreased to 1±1 at 48 hours. No postoperative opioid administration was documented in the reviewed medical records. Early mobilization occurred at 12 hours, with progressive functional recovery and a mean hospital stay of four days. No major complications or signs of local anesthetic systemic toxicity were observed. Patient satisfaction was high (93.3% satisfied or very satisfied).

Conclusion

This retrospective case series describes the implementation of a standardized periarticular LIA protocol associated with low early postoperative pain scores and favorable short-term recovery outcomes within a multimodal and ERAS-based pathway. Due to the retrospective design and absence of a control group, comparative efficacy cannot be established, and findings should be interpreted as descriptive and hypothesis-generating. Prospective controlled studies are required to evaluate its effectiveness against contemporary motor-sparing regional anesthesia techniques.

## Introduction

Total knee arthroplasty (TKA) is an established treatment for advanced knee osteoarthritis and generally improves pain, physical function, and health-related quality of life when conservative treatment fails [1]. However, the immediate postoperative period remains challenging because pain after TKA is often intense, multifactorial, and closely linked to delayed rehabilitation and poorer early patient experience.

Inadequate control of acute postoperative pain may delay mobilization, increase opioid consumption, prolong hospital stay, and contribute to persistent postsurgical pain beyond the early recovery period [2]. For this reason, multimodal analgesia has become a central component of enhanced recovery pathways after TKA.

Peripheral nerve blocks, particularly femoral nerve block and adductor canal-based techniques, provide effective analgesia after TKA, but their use must be balanced against transient motor weakness, increased logistic complexity, and - especially with femoral-based continuous blocks - a documented risk of falls [3-5]. These limitations have encouraged interest in analgesic approaches that preserve quadriceps function while still targeting the multiple periarticular pain generators involved in knee arthroplasty.

Local infiltration analgesia (LIA), first popularized as a systematic periarticular infiltration technique in hip and knee arthroplasty, aims to deliver high local analgesic concentrations directly into the tissues injured during surgery without producing major motor blockade [6,7]. Reported benefits include reduced early pain, reduced opioid use, and facilitation of early rehabilitation, although protocol heterogeneity and mixed comparative results continue to limit consensus.

The aim of this study was to describe early postoperative outcomes following a standardized periarticular local infiltration analgesia (LIA) protocol in primary TKA. The primary objective was to evaluate early postoperative pain trajectories using visual analogue scale (VAS) scores and in-hospital opioid exposure. Secondary objectives included early mobilization milestones, knee range of motion at discharge, length of hospital stay, perioperative safety, and patient satisfaction. This study is designed as a descriptive, hypothesis-generating case series intended to report real-world outcomes rather than establish comparative efficacy.

## Materials and methods

Study design and setting

This retrospective single-center case series was conducted in the Department of Trauma and Orthopedic Surgery at Military Hospital Avicenne, Marrakech, Morocco. This study was approved by the institutional review board, and the requirement for informed consent was waived due to its retrospective design. Medical records were reviewed for consecutive patients who underwent primary TKA between January 2025 and June 2025 and received standardized intraoperative periarticular LIA.

Participants

Eligible patients underwent primary TKA for symptomatic knee osteoarthritis with a validated surgical indication. The inclusion criteria for the study were primary TKA, spinal anesthesia according to institutional practice, availability of complete perioperative records, and at least six months of postoperative follow-up. The criterion of follow-up after six or more months was used primarily for safety surveillance and identification of delayed complications; no medium- or long-term functional outcomes were analyzed in this study. Exclusion criteria were unicompartmental arthroplasty, revision TKA, documented allergy to local anesthetics, or missing essential data. All consecutive eligible patients during the study period were included to minimize selection bias. A total of 30 patients met these criteria and were included in the analysis.

Perioperative management and infiltration protocol

All procedures were performed under spinal anesthesia. Intrathecal opioid administration was not routinely used. A pneumatic tourniquet was used in all cases. The surgeon performed periarticular infiltration in three standardized stages targeting the posterior capsule and gastrocnemius region before implantation, deep periarticular structures after implantation, and the subcutaneous layer after wound closure. The infiltration protocol was developed and implemented as a standardized institutional practice by the surgical team and was applied consistently across all patients without intentional variation in volumes or composition.

The injectate consisted of bupivacaine 0.5% (total dose 150 mg) and lidocaine 2% (total dose 180 mg), diluted in saline to achieve a high-volume, low-concentration solution (final bupivacaine concentration approximately 0.125%). No adjuncts such as epinephrine or ketorolac were used. This approach was chosen to optimize tissue diffusion and periarticular coverage rather than relying on high drug concentration alone.

All patients received standard perioperative multimodal analgesia according to institutional practice, including paracetamol and nonsteroidal anti-inflammatory drugs (unless contraindicated). Additional intraoperative anesthetic agents were administered at the discretion of the anesthesiologist. No standardized postoperative opioid protocol was routinely implemented.

Postoperative rehabilitation followed an early mobilization pathway: patients were encouraged to sit at the bedside on the day of surgery or postoperative day 1, with progressive assisted ambulation and physiotherapy sessions conducted daily. Discharge was based on clinical recovery, pain control, and functional mobility.

Outcome measures

The primary endpoint was early postoperative analgesia, assessed using the visual analog scale (VAS), a widely used instrument ranging from 0 (no pain) to 10 (worst imaginable pain) [8]. VAS scores were collected at six, 12, 24, and 48 hours after surgery. Secondary outcomes were postoperative analgesic consumption, time to first bedside mobilization, time to active knee mobilization, time to assisted ambulation, knee range of motion at discharge, length of stay, complications related to the procedure or local anesthetics, and patient-reported satisfaction.

Postoperative opioid use was assessed through a review of multiple sources, including post-anesthesia care unit (PACU) charts, medication administration records, and nursing notes. Both strong and weak opioids (including tramadol) were considered. The absence of opioid use was defined as no documented administration in the reviewed records.

Data collection

Data were retrospectively abstracted by the study investigators using a standardized data extraction approach. Sources included electronic medical records, anesthetic charts, medication administration records, and nursing documentation. Where discrepancies existed, records were cross-checked to improve data accuracy. Missing data were handled using a complete-case analysis approach and are reported where applicable.

Statistical analysis

Because the study had no control group and included a small number of patients, the analysis was primarily descriptive. Continuous variables are reported as mean±standard deviation or median with interquartile range when appropriate depending on data distribution. Categorical variables are reported as counts and percentages. Pain evolution over time was summarized numerically and graphically. No inferential statistical comparisons were performed, in keeping with the descriptive and hypothesis-generating nature of the study.

## Results

Patient characteristics

The study population comprised 30 patients with a mean age of 72.2±7.3 years (range, 59-89 years). Baseline characteristics, including body mass index (BMI) and American Society of Anesthesiologists (ASA) physical status, are reported in Table 1 to better characterize the study population and perioperative risk profile. Women accounted for 73.3% of the cohort. Hypertension, obesity, and rheumatic disease were the most frequently recorded comorbidities. All baseline variables were available for the included patients, with no missing data requiring imputation. Baseline demographic and clinical characteristics are summarized in Table 1.

**Table 1:** Baseline demographic and clinical characteristics Percentages for comorbidities are not mutually exclusive as patients may present with more than one condition.

Variable	Value
Age, years	72.2±7.3 (range 59-89)
Female sex	22 (73.3%)
Male sex	8 (26.7%)
Right knee	17 (56.7%)
Left knee	13 (43.3%)
Hypertension	15 (50.0%)
Diabetes mellitus	8 (26.7%)
Obesity	14 (46.7%)
Cardiac disease	5 (16.7%)
Rheumatic disease	13 (43.3%)
Previous knee surgery	7 (23.3%)
History of knee trauma	3 (10.0%)

Perioperative findings

All patients underwent primary TKA under spinal anesthesia, and all received the standardized periarticular LIA protocol. No deviations from the institutional perioperative protocol were recorded in the reviewed medical records (Table 2).

**Table 2:** Perioperative findings Operative time and blood loss are reported as mean±standard deviation (SD). Tourniquet use was uniform across all cases.

Variable	Value
Operative time	90±15 min (mean±SD)
Blood loss	350±50 mL (mean±SD)
Tourniquet use	30 (100%)

Intraoperative infiltration protocol

The perioperative infiltration protocol was performed in three stages. The protocol was applied consistently across all patients as part of an institutional standardized technique developed by the surgical team, with no intentional variation in composition or dosing (Table 3).

**Table 3:** Standardized periarticular infiltration protocol Total planned dose per patient: bupivacaine 150 mg, lidocaine 180 mg, total infiltrated volume 120 mL.

Stage	Injection site(s)	Injected mixture	Volume
Stage 1: before implant placement	Posterior capsule and gastrocnemius region	10 mL bupivacaine 0.5%+3 mL lidocaine 2%+27 mL saline	40 mL
Stage 2: after implant placement	Infrapatellar fat pad, pes anserinus region, and synovial recesses	10 mL bupivacaine 0.5%+3 mL lidocaine 2%+27 mL saline	40 mL
Stage 3: after wound closure	Subcutaneous plane	10 mL bupivacaine 0.5%+3 mL lidocaine 2%+27 mL saline	40 mL

The final diluted concentration corresponded to approximately 0.125% bupivacaine and low-dose lidocaine intended for combined early and intermediate analgesic effects. No adjuncts (epinephrine or ketorolac) were used.

Pain and analgesic outcomes

**Figure 1:**
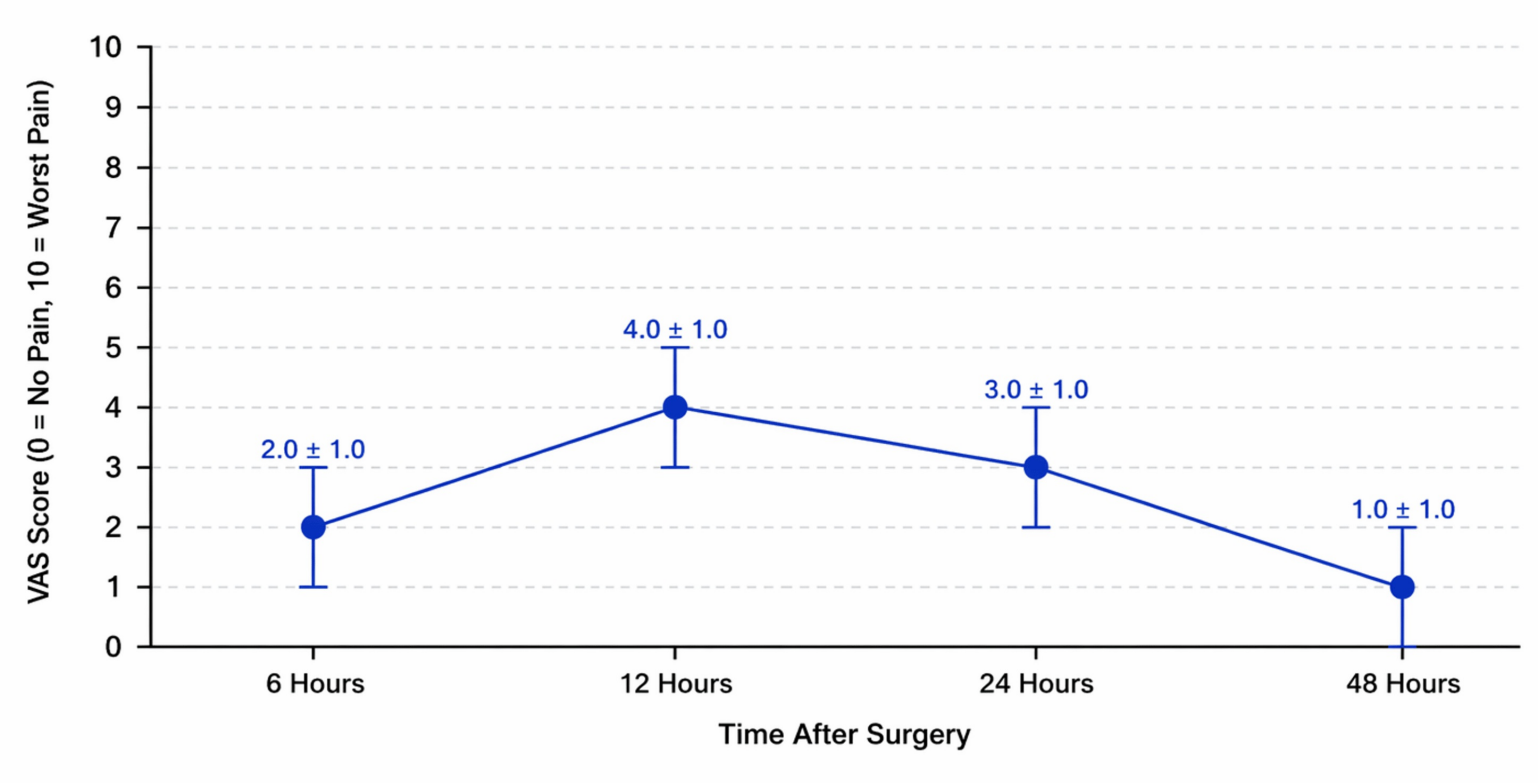
Postoperative Pain Trajectory Measured by Visual Analog Scale (VAS) After Primary Total Knee Arthroplasty. Postoperative pain trajectory measured by visual analog scale (VAS, 0-10) at six, 12, 24, and 48 hours after primary total knee arthroplasty. Data are presented as mean±standard deviation (SD).

Postoperative pain, assessed using the visual analog scale (VAS, 0-10), demonstrated a progressive decrease over the first 48 hours following surgery. At six hours postoperatively, the mean VAS score was 2±1 for all patients (n=30). At 12 hours, the mean VAS increased to 4±1, followed by a reduction to 3±1 at 24 hours, and further decreased to 1±1 at 48 hours (Figure 1). Although no formal comparative statistical testing was performed, this trend suggests a clinically meaningful reduction in pain intensity over time during the early postoperative period.

Perioperative and postoperative outcomes were systematically extracted from electronic medical records and anesthesia/surgical charts using a predefined data collection form. Intraoperative variables (operative time, blood loss, and tourniquet use) were obtained from operative reports, while postoperative outcomes (VAS scores, mobilization milestones, analgesic consumption, complications, and length of stay) were retrieved from nursing charts, physiotherapy records, and medication administration records. Patient satisfaction was recorded from standardized discharge documentation. Continuous variables are reported as mean±standard deviation, and categorical variables as counts and percentages.

Table 4 presents the complete set of perioperative and postoperative outcomes.

**Table 4:** Perioperative and postoperative outcomes Continuous variables are presented as mean±standard deviation (SD), and categorical variables as number/total (%). VAS are expressed on a 0-10 scale. Bedside mobilization, active knee mobilization, and assisted ambulation were recorded as separate recovery milestones in the source records. VAS: Visual Analog Score.

Parameter	Result
Operative time	90±15 min
Estimated blood loss	350±50 mL
Tourniquet use	30/30 (100%)
VAS at 6 h	2.0±1.0 (VAS units, 0-10 scale)
VAS at 12 h	4.0±1.0 (VAS units, 0-10 scale)
VAS at 24 h	3.0±1.0 (VAS units, 0-10 scale)
VAS at 48 h	1.0±1.0 (VAS units, 0-10 scale)
Postoperative opioid use	0/30 (0%)
First bedside mobilization	12±2 h
Active knee mobilization	48±1 h
Assisted ambulation	3±1 days
Knee flexion at discharge	90±10°
Length of hospital stay	4±1 days
Major complications	0/30 (0%)
Patient satisfaction (satisfied/very satisfied)	28/30 (93.3%)

Functional recovery

The first bedside mobilization occurred after a mean of 12 hours. Structured active knee mobilization was initiated after 48±1 hours, assisted ambulation resumed after 3.5±0.5 days, and mean knee flexion at discharge was 90±10°. The mean length of stay was four days.

Safety and patient satisfaction

No major complications were reported. No superficial hematoma, surgical-site infection, allergic reaction, delayed wound healing, or clinical sign of local anesthetic systemic toxicity was recorded. Twenty patients (66.7%) were very satisfied, eight (26.7%) were satisfied, and two (6.7%) were slightly satisfied with postoperative pain management. Table 5 presents the distribution of patient satisfaction categories.

**Table 5:** Patient satisfaction categories

Category	Value
Very satisfied	20 (66.7%)
Satisfied	8 (26.7%)
Slightly satisfied	2 (6.7%)
Not satisfied	0 (0%)

## Discussion

This retrospective case series suggests that a standardized periarticular LIA protocol can be integrated safely into primary TKA performed under spinal anesthesia and may provide low early postoperative pain scores, negligible documented opioid exposure, and acceptable early rehabilitation milestones.

The postoperative pain trajectory demonstrated an initial increase at 12 hours followed by a progressive decline up to 48 hours. This pattern is consistent with the expected early postoperative inflammatory peak after total knee arthroplasty rather than a sustained analgesic effect alone [9-13].

At the same time, the comparative literature remains heterogeneous. Some trials have suggested that continuous femoral nerve block may provide equal or even superior early analgesia and functional performance in selected settings [11], whereas others have found that periarticular infiltration offers analgesia comparable to femoral block while being cheaper, simpler, and less likely to impair motor function [10,12]. This inconsistency is not surprising given the marked variation in drug mixtures, injection sites, timing, systemic co-analgesia, and rehabilitation protocols across studies.

The present study does not aim to demonstrate analgesic superiority but rather to describe real-world postoperative pain evolution following a standardized periarticular infiltration protocol.

The motor-sparing rationale for periarticular infiltration remains clinically attractive. Contemporary postoperative pathways increasingly favor techniques that preserve quadriceps strength, such as adductor canal-based analgesia and posterior capsule blocks, rather than traditional femoral-based strategies [14-16]. Periarticular infiltration fits well within this trend because it is surgeon-delivered, technically straightforward, and does not require dedicated postoperative catheter management.

In the present study, the periarticular infiltration protocol was implemented as an institutional standardized protocol applied to all consecutive eligible patients during the study period. The injectate composition, volumes, and three-stage technique were defined by the senior orthopedic surgical team and consistently applied without intra-cohort variation, under routine institutional practice conditions.

The relatively high total infiltration volume (120 mL) and the diluted local anesthetic concentrations (bupivacaine 0.125% final concentration and lidocaine 0.05% final concentration) are consistent with previously described periarticular infiltration strategies in TKA designed to maximize tissue spread while maintaining safe systemic plasma levels. These concentrations remain within accepted safety limits when used in combination and when distributed across multiple anatomical planes.

The analgesic effect observed cannot be attributed solely to the systemic duration of action of local anesthetics. Rather, periarticular infiltration likely provides prolonged analgesia through sustained peripheral nociceptor modulation, inhibition of local inflammatory mediators, and prolonged tissue reservoir effect, particularly in periarticular fat, capsule, and synovial tissues, where drug diffusion and reabsorption are delayed. This mechanism has been described in experimental and clinical studies of local infiltration analgesia.

Our protocol differed from many published regimens by using bupivacaine and lidocaine diluted in saline without documented addition of ketorolac or epinephrine. Despite this relatively simple mixture, no clinical toxicity and no major wound-related complications were observed. The absence of documented opioid use is notable, although it should be interpreted cautiously because chart-based retrospective studies may underreport rescue medication administered outside standardized forms.

Importantly, the present study does not include a control group and therefore cannot establish superiority or infer comparative effectiveness versus femoral nerve block, adductor canal block, intrathecal morphine, or other enhanced recovery after surgery (ERAS)-based multimodal strategies. The VAS values presented represent isolated descriptive postoperative trajectories and should be interpreted within the context of expected outcomes after TKA under modern multimodal analgesia. Direct comparison with “standard care” or alternative regional techniques is not possible from this dataset.

The study has several limitations. First, the sample size was small. Second, the absence of a control group precludes any direct comparison with femoral block, adductor canal block, intrathecal morphine, or systemic analgesia alone. Third, this was a single-center retrospective analysis, which limits external validity and introduces the possibility of incomplete documentation. Fourth, satisfaction and functional outcomes were not assessed with validated postoperative recovery scales. Fifth, potential information bias related to retrospective chart review may have led to underreporting of rescue opioid administration and minor postoperative adverse events. Sixth, although consecutive patients were included, residual selection bias cannot be completely excluded. These limitations mean that the present results should be interpreted as hypothesis-generating rather than definitive.

Nevertheless, the study offers useful pragmatic information. It documents a feasible periarticular infiltration protocol, provides short-term recovery data from a real-world practice setting, and supports the broader evidence synthesis suggesting that LIA can contribute meaningfully to multimodal pain control after TKA [17,18]. Future work should compare standardized LIA regimens against contemporary motor-sparing block strategies in larger prospective cohorts or randomized studies.

## Conclusions

This retrospective case series describes the implementation of a standardized periarticular local infiltration analgesia protocol in primary TKA performed under spinal anesthesia within a multimodal and ERAS-based perioperative pathway. The technique was associated with low early postoperative pain scores, early mobilization, and satisfactory short-term recovery outcomes in this 30-patient cohort. No postoperative opioid administration was documented in the reviewed medical records.

However, due to the retrospective design and absence of a control group, comparative effectiveness cannot be established, and no conclusions regarding superiority over other analgesic strategies can be drawn. In addition, potential underreporting of opioid administration and lack of validated functional outcome measures limit the strength of the findings.

Therefore, these results should be interpreted as descriptive and hypothesis-generating, supporting the feasibility of the technique in routine clinical practice while highlighting the need for prospective, controlled studies with standardized functional and patient-reported outcome measures to define its role among contemporary multimodal and motor-sparing analgesic strategies.
